# Supplementary material for: ATP Inhibits Breast Cancer Migration and Bone Metastasis through Down-Regulation of CXCR4 and Purinergic Receptor P2Y11
Source: Cancers (Basel). 2021 Aug 26;13(17):4293. doi: 10.3390/cancers13174293 (PMC8428338; doi:10.3390/cancers13174293)
Supplement: Supplementary file 1 [file cancers-13-04293-s001.zip › cancers-1301769-supplementary.pdf]

Supplementary Materials

ATP Inhibits Breast Cancer Migration and Bone Metastasis Through Down-Regulation of CXCR4 and Purinergic Receptor P2Y11

Xiaowen Liu, Manuel A. Riquelme, Yi Tian, Francisca M. Acosta, Sumin Gu and Jean X. Jiang

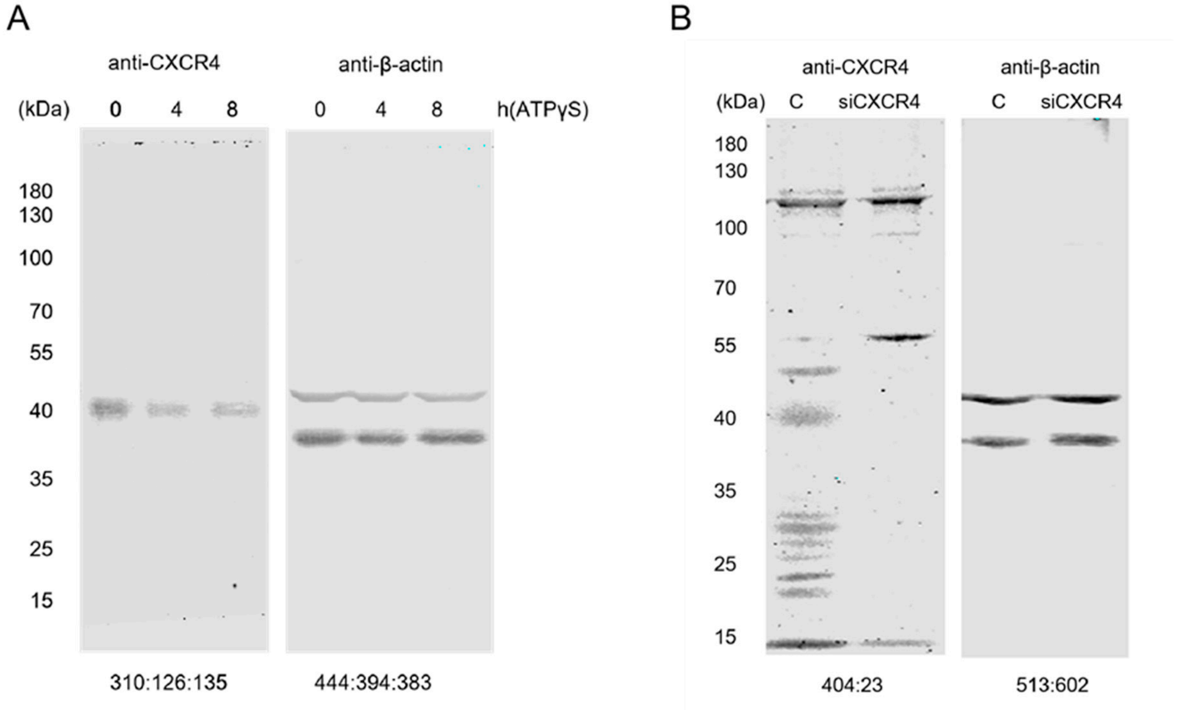

Figure S1. Western blot data. (**A**) MDA-BoM-1833 cells were treated with 100 μM ATPγS for 4 or 8 h and level of CXCR4 protein was determined by Western blotting with anti-CXCR4 or β-actin antibody (upper band is β-actin and lower band is GAPDH). (**B**) MDA-BoM-1833 cells were transfected with CXCR4 siRNA and 48 h after transfection, Western blotting was performed with anti-CXCR4 or β-actin antibody (upper band is β-actin and lower band is GAPDH).

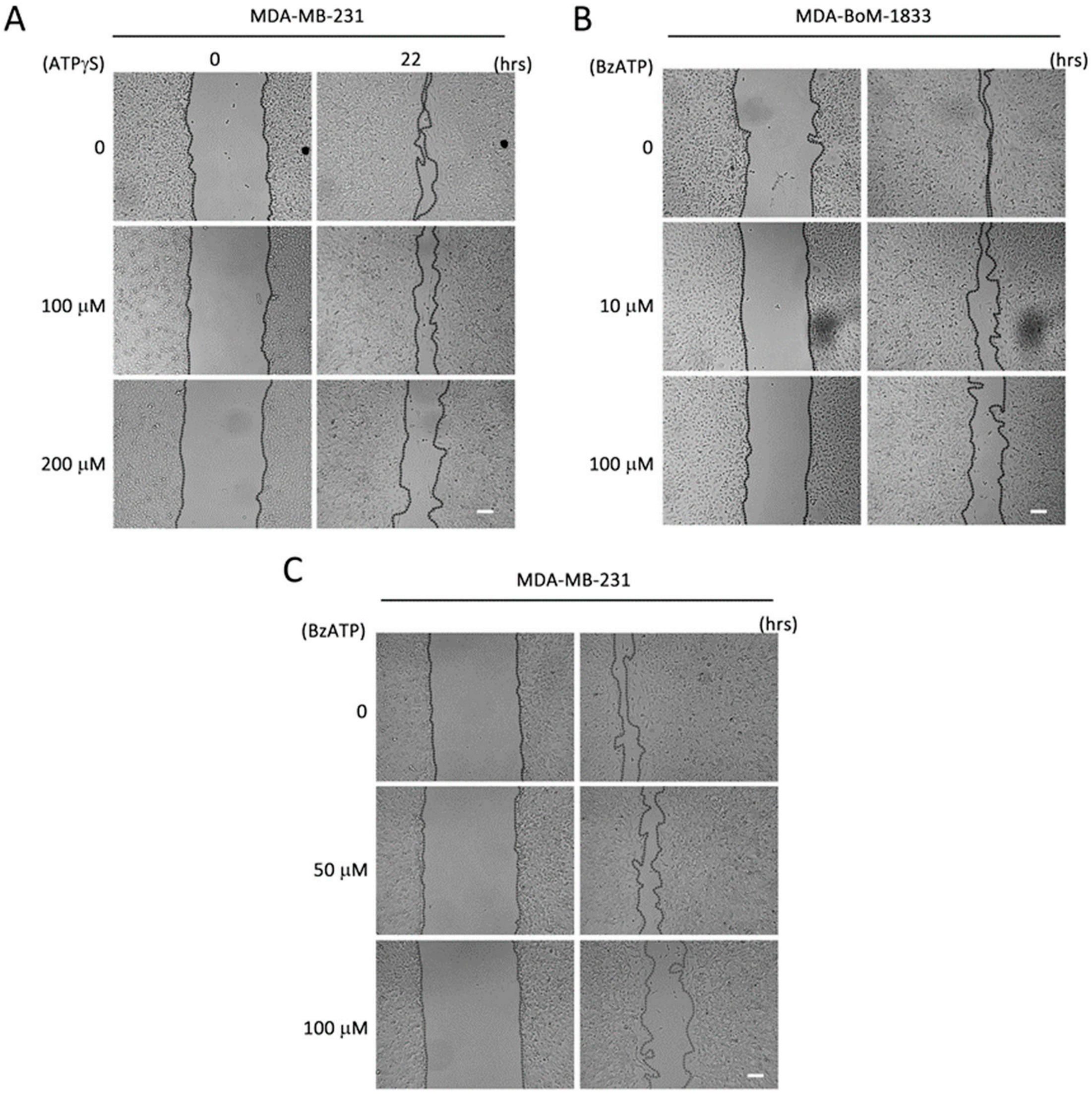

Figure S2. Representative wound-healing assay images. (**A**) Representative scratch assay pictures of wound healing assay which was performed in MDA-MB-231 cells after treatment with various concentrations of ATPγS (quantified data showed in Figure 1D). (**B**) Representative scratch assay pictures of wound healing assay which was performed in MDA-BoM-1833 cells after treatment with various concentrations of BzATP (quantified data showed in Figure 2A). (**C**) Representative scratch assay pictures of wound healing assay which was performed in MDA-BoM-1833 cells after treatment with various concentrations of BzATP (quantified data showed in Figure 2D).
